# A Case of Poisoning by Arsenious Acid

**Published:** 1853-06

**Authors:** J. Bryant Smith

**Affiliations:** Louisville, Ky.


*A Case of Poisoning by Arsenious Acid.* Reported by J. Bryant Smith, M. D., of Louisville, Ky.—During the month of February last, a negro woman and her child, residing in this city, were suddenly attacked with symptoms of poisoning after drinking some tea which had been prepared by some members of the family. The physician who was called in attendance, not suspecting poison, administered some laxative oil, which produced no effect. The child and mother were vomiting continually, complained of burning pain in the epigastric region, the pulse feeble and fluttering, the extremities cold, face anxious and debilitated.

During the day following the child died, and a post-mortem was made by the coroner of the city, and the stomach of the child removed. The case having come under the observation of Dr. Lyle, of this city, he obtained the coffee-pot with its contents, from which the patients had drank, and brought it to the laboratory of the University of Louisville, for analysis. The vessel contained about a quart of tea, with a quantity of white sediment, nearly half an ounce in weight, which, when placed on hot coals, emitted the peculiar alliaceous odor of arsenic. On examining the tea by Marsh's test, it was found to give unmistakeable evidence of the presence of arsenic, and appeared to be nearly, if not quite, a saturated solution of arsenious acid.

The coroner having brought to Prof. Silliman the stomach removed from the child, it was subjected to the process which, as it is interesting to the physician and easy of application in similar cases, may prove useful to the readers of your Journal.

This portion of the viscera was tied before removal from the body, at both the œsophageal and pyloric orifices, and the contents thereby secured. On removing these ligatures, there flowed about four fluid ounces of a dark, coffee-colored fluid, having the flavor of some essential oil. This fluid gave, with the tests to be described, no evidence of arsenic. The mucous lining of the stomach was, in some spots, slightly ecchymosed, but otherwise presented no abnormal appearance. A portion of it was cut into small pieces, placed in a porcelain evaporating dish, and treated with a very few drops of sulphuric acid and a drop of nitric acid, and heated. By this means the organic matter, which, when present, makes the detection of arsenic difficult, is destroyed, and the insoluble arsenious acid, if present, is converted into soluble arsenic acid; the material soon becomes reduced to a coaly mass, it is heated then until the sulphuric acid is driven off, allowed to cool, and then treated with water to remove the arsenic acid, if present. This liquid thus obtained is filtered and treated with a current of sulphuretted hydrogen gas obtained in the usual way from sulphuret of iron and sulphuric acid.

By this process, conducted in the case of the stomach of the child, we obtained, after a time, a small yellow precipitate; this, if arsenic had been present in the solution, would have been the "yellow sulphuret of arsenic" or orpiment. But in acid solution, sulphuretted hydrogen may precipitate sulphur, which may be mistaken for orpiment. If the precipitate is very small, as it was in the present case, it is boiled to facilitate its being separated, filtered from the liquid, and then treated with aqua ammoniæ, which dissolves it, if it be orpiment, but has no action on sulphur. The solution in ammonia is now evaporated to dryness, and the residue redissolved in hydrochloric acid. We have now a solution containing all the arsenic which may have been present in the portion of stomach or other viscera under examination, and which we can subject to the appropriate tests for the detection of arsenic. The one which in this case we employed first was one known as "Reinsch's," which consists in boiling in the hydrochloric acid solution a strip or two of perfectly clean bright copper. If arsenic be present in the solution, these strips are soon coated with a grey deposit of metallic arsenic. In the case under examination, on the slips of copper, in the course of a few minutes, there was an evident blackening, sufficient to render the presence of arsenic in the solution certain. To corroborate this test, recourse was next had to "Marsh's" test, which consists in adding the solution suspected to contain arsenic to a flask or bottle in which are placed the materials for generating hydrogen gas, namely, zinc and sulphuric acid. Pure hydrogen gas, when burning, is entirely converted into water, and hence there is deposited on any cold surface held over it, only this compound; but the presence of arsenic in the generating flask, gives rise to the production of "arsenuretted hydrogen," which produces a peculiar change in the flame, and an evolution of white clouds of arsenious acid. If over this flame, and about its centre, is held a piece of cold porcelain or any white dish, there will be deposited a brownish-black "tache" or spot, with a metallic luster. There is only one other metal with which this is liable to be confounded, and that is antimony, which produces like results under similar circumstances. It is, however, more black and has less luster. The two are also easily distinguishable. Exposed to the vapor of iodine in a small crucible, the antimony spots turn reddish orange, while arsenical spots appear orange-yellow, and soon entirely vanish.

Exposed for a moment to the vapor of chlorine gas obtained from ordinary bleaching powder, (hydrochlorite of lime,) in a capsule, the spots of both metals disappear. If a drop of nitrate of silver be then allowed to fall on the surface of the vessel on which the spots were, if arsenic be present, (in the form of chloride now,) there will be a brick red stain visible, amounting to a precipitate if the metal existed in any quantity, while antimony produces no such results. These distinctions are conclusive. In the case which was examined in this way, we detected in the stomach and liver of the child perfectly decisive evidence of arsenic by both of these tests.

During the progress of this investigation, an old man, who had also drank of the tea, had died, and as poisoning was suspected, he was disinterred and the body opened in presence of Dr. Silliman and myself, by Dr. Lyle. The liver and vessels were very much engorged ; the blood not coagulated ; the stomach and liver were removed and taken to the laboratory, and subjected to the same process of investigation before described. In both the evidences of arsenic were as conclusive as in the case of the child. The mother, who at the time was nursing, recovered, but complained of her infant being sickened by her milk and refusing to nurse. A portion of the milk was sent us for analysis ; it was very dark colored, and gave, by the same test, evidence of the presence of arsenic. I am not aware that this poison has ever been detected before in this secretion, and it may be the happy accident of lactation which saved the life of the mother, in opening thus a channel through which the poison was discharged from the system.

The criminal part of this case rests in a negro man who had for a long time professed friendship for the family, and under such profession had become quite intimate. The morning on which the poison was administered he made some base offers to the woman and her sisters, which were repulsed, when, in revenge, he infused arsenious acid into the tea. The evidence which was given in this case was deemed sufficient to remand the prisoner for trial in the following term of the Circuit Court of Jefferson.

It seems to me that there ought to be in every State some legislation whereby the sale of arsenic may be restricted to druggists properly authorized, and that such druggists should be compelled to keep a register, in which every sale of arsenic should be entered, with the name, residence, and occupation of the buyer and the purposes for which obtained. Under such vigilant restrictions no person would dare to purchase arsenical poison except for lawful purposes.—*Buffalo Med. Journal.*

